# Minimally invasive distal pancreatectomy: a case-matched cost-analysis between robot-assisted surgery and direct manual laparoscopy

**DOI:** 10.1007/s00464-021-08332-1

**Published:** 2021-02-03

**Authors:** Gregorio Di Franco, Andrea Peri, Valentina Lorenzoni, Matteo Palmeri, Niccolò Furbetta, Simone Guadagni, Desirée Gianardi, Matteo Bianchini, Luca Emanuele Pollina, Franca Melfi, Domenica Mamone, Carlo Milli, Giulio Di Candio, Giuseppe Turchetti, Andrea Pietrabissa, Luca Morelli

**Affiliations:** 1 General Surgery Unit, Department of Translational Research and New Technologies in Medicine and Surgery, University of Pisa, Via Paradisa 2, 56125 Pisa, Italy; 2 Department of Surgery, Fondazione IRCCS Policlinico San Matteo, Pavia, Italy; 3 Institute of Management, Scuola Superiore Sant’Anna, Pisa, Italy; 4 Second Division of Surgical Pathology, University Hospital of Pisa, Pisa, Italy; 5 Multidisciplinary Center of Robotic Surgery, University Hospital of Pisa, Pisa, Italy; 6 Pharmaceutical Unit, Medical Device Management, University Hospital of Pisa, Pisa, Italy; 7 Board of Directors, University Hospital of Pisa, Pisa, Italy; 8 EndoCAS (Center for Computer Assisted Surgery), University of Pisa, Pisa, Italy

**Keywords:** Robotic distal pancreatectomy, Laparoscopic distal pancreatectomy, Costs’ analysis

## Abstract

**Background:**

Few studies have reported a structured cost analysis of robotic distal pancreatectomy (RDP), and none have compared the relative costs between the robotic-assisted surgery (RAS) and the direct manual laparoscopy (DML) in this setting. The aim of the present study is to address this issue by comparing surgical outcomes and costs of RDP and laparoscopic distal pancreatectomies (LDP).

**Methods:**

Eighty-eight RDP and 47 LDP performed between January 2008 and January 2020 were retrospectively analyzed. Three comparable groups of 35 patients each (Si-RDP-group, Xi-RDP group, LDP-group) were obtained matching 1:1 the RDP-groups with the LDP-group. Overall costs, including overall variable costs (OVC) and fixed costs were compared using generalized linear regression model adjusting for covariates.

**Results:**

The conversion rate was significantly lower in the Si-RDP-group and Xi-RDP-group: 2.9% and 0%, respectively, versus 14.3% in the LDP-group (*p* = 0.045). Although not statistically significant, the mean operative time was lower in Xi-RDP-group: 226 min versus 262 min for Si-RDP-group and 247 min for LDP-group. The overall post-operative complications rate and the length of hospital stay (LOS) were not significantly different between the three groups. In LDP-group, the LOS of converted cases was significantly longer: 15.6 versus 9.8 days (*p* = 0.039). Overall costs of LDP-group were significantly lower than RDP-groups, (*p* < 0.001). At multivariate analysis OVC resulted no longer statistically significantly different between LDP-group and Xi-RDP-group (*p* = 0.099), and between LDP-group and the RDP-groups when the spleen preservation was indicated (*p* = 0.115 and *p* = 0.261 for Si-RDP-group and Xi-RDP-group, respectively).

**Conclusions:**

RAS is more expensive than DML for DP because of higher acquisition and maintenance costs. The flattening of these differences considering only the variable costs, in a high-volume multidisciplinary center for RAS, suggests a possible optimization of the costs in this setting. RAS might be particularly indicated for minimally invasive DP when the spleen preservation is scheduled.

Since the description of the first minimally invasive distal pancreatectomy (MIDP) in 1994 [1], the use of the laparoscopic technique for distal pancreatectomy has progressively increased. In fact, MIDP is now considered the standard approach for benign and premalignant pancreatic tumors, especially in centers with high experience with pancreatic surgery [2]. Moreover, also for malignant pancreatic tumors, the MIDP seems to be equivalent to the traditional open approach in terms of oncological outcomes (i.e., R0 resection, resection margins, harvested lymph nodes, 30-day mortality, disease-free survival, and overall survival) [2]. However, although MIDP has been associated to clear advantages over the open approach in terms of pain, intraoperative blood loss, and length of hospital stay (LOS) [3–5], it is still performed with a traditional approach in most hospitals, because of the deep anatomical location of the pancreas and the complexity of distal pancreatectomy. In this regard, the widespread adoption of robotic-assisted surgery (RAS) also in pancreatic surgery could favor the increasing of MIDP.

Robotic distal pancreatectomy (RDP) seems also to be associated with an additional positive impact on the clinical outcomes as well, with a reduction of conversion rates, higher spleen preservation rates, and a shorter LOS [2, 6, 7]. A potential barrier to the diffusion of the robotic approach could be its economic impact [8]; indeed, it is well known that RAS is generally associated with higher costs in comparison to direct manual laparoscopy (DML), also for RDP, as data emerged from previous studies, although controversial, are almost unanimously oriented toward higher cost for RAS [3, 9–14]. However, despite at the beginning of the experience with the robotic platforms the economic analyses were strongly against the use of the da Vinci Surgical System, more recent data seem to have opened new perspectives in this regard [15]. Therefore, the aim of our study is to perform a structured cost-analysis, including the short-term outcomes, of distal pancreatectomy performed with RAS, with both the da Vinci Si and Xi in a multidisciplinary robotic center, and with DML.

## Materials and methods

From January 2008 to January 2020, 88 patients underwent RDP at the Multidisciplinary Center of Robotic Surgery of Pisa, performed by the 1st General Surgery Unit, and were included in the present study. All patients were operated by two surgeons (AP and LM), both with high experience of pancreatic surgery (> 300 procedures each) and minimally invasive surgery (both laparoscopic and robotic surgery, > 300 procedures each), and both trained in the high-volume center of Pisa (> 100 pancreatic resections/year).

The choice of the operative technique (laparoscopic or robot-assisted) was at the discretion of the operating surgeon and based on the robotic platform availability, being that the second one has been the preferred approach in the last 8 years.

The RDPs were performed with the da Vinci Si platform until December 2014. Since the introduction of the da Vinci Xi at our center, in January 2015, the choice of the platform of da Vinci Surgical System (da Vinci Si or da Vinci Xi) depended on their availability. In detail, 47 patients underwent RDP with the da Vinci Si (Si-RDP-group) and 41 patients with the da Vinci Xi (Xi-RDP-group). In the same period 47 patients underwent laparoscopic distal pancreatectomy (LDP-group) for the same indications by the same two surgeons (AP and LM). In order to compare outcomes and costs among the three groups minimizing possible biases deriving from treatment allocation, three comparable groups were obtained matching 1:1 the two robotic groups with the LDP-group. The following patients’ characteristics were considered for the matching: age, gender, Body Mass Index (BMI), and American Society of Anesthesiology (ASA) score. After matching, the final population included 105 patients, equally divided between each group: Si-RDP-group (*n* = 35), Xi-RDP group (*n* = 35), and LDP-group (*n* = 35).

Data on patients’ preoperative characteristics, surgical procedures, post-operative course, follow-up and resources used (i.e., associated to operative time, length of stay, etc.) were retrospectively reviewed and analyzed, from a prospectively collected database.

The preoperative workup included abdominal ultrasonography, abdomen CT and/or MRI. Indications for minimally invasive approach were neoplasms < 10 cm with benign or borderline features on cross-sectional imaging or adenocarcinomas, similarly to other teams [11, 16].

### Surgical procedures

Laparoscopic and robotic distal pancreatectomies were performed as already described [17]. Splenic preservation was performed only in case of presumed benign or premalignant lesions and pursued according to the splenic vessels conservation technique described by Kimura et al. [18]. Splenic conservation performed by sacrificing the splenic vessels (Warshaw technique [19]) was not performed in our series. In both laparoscopic or robotic techniques, parenchymal transection and closure was carried out using electrocautery and the stump was over sewn with intracorporeal suturing and knotting, or with an endostapler.

*Laparoscopic DP* The patient is placed in the supine or left sided position, dependent upon the tumor site, with both arms along the sides of the body, and tilted in partial reverse Trendelenburg position. Four/five ports are used (2/3, 5 mm; 2, 12 mm). After the establishment of pneumoperitoneum and the ports placement, an abdominal exploration is performed. The lesser sac is then entered through the greater gastrocolic omentum. The splenic flexure of the colon is mobilized, if necessary. An intra-operative diagnostic ultrasound with a laparoscopic probe is always performed to evaluate the pancreatic lesion and its correct position. The superior and inferior borders of the pancreas are defined and the pancreas is transected using a stapler (ETS Flex 45 Endoscopic Articulating Staple, Johnson & Johnson, USA). The distal transected pancreas is gently lifted, and a medial-to- lateral dissection is started. In case of splenic conservation, the splenic vein and artery are skeletonized from the isthmus toward the splenic hilum. This manoeuvre allows both the lymphadenectomy and a step-by-step division of all the branches coming from the splenic vessels. In case of DP with splenectomy, first the splenic artery and then the vein are divided after the transection of the pancreas. This step is followed by a medial-to-lateral dissection posterior to the splenic vein along the retroperitoneal plane. Two drains are left near the pancreatic stump.

**Fig. 1:**
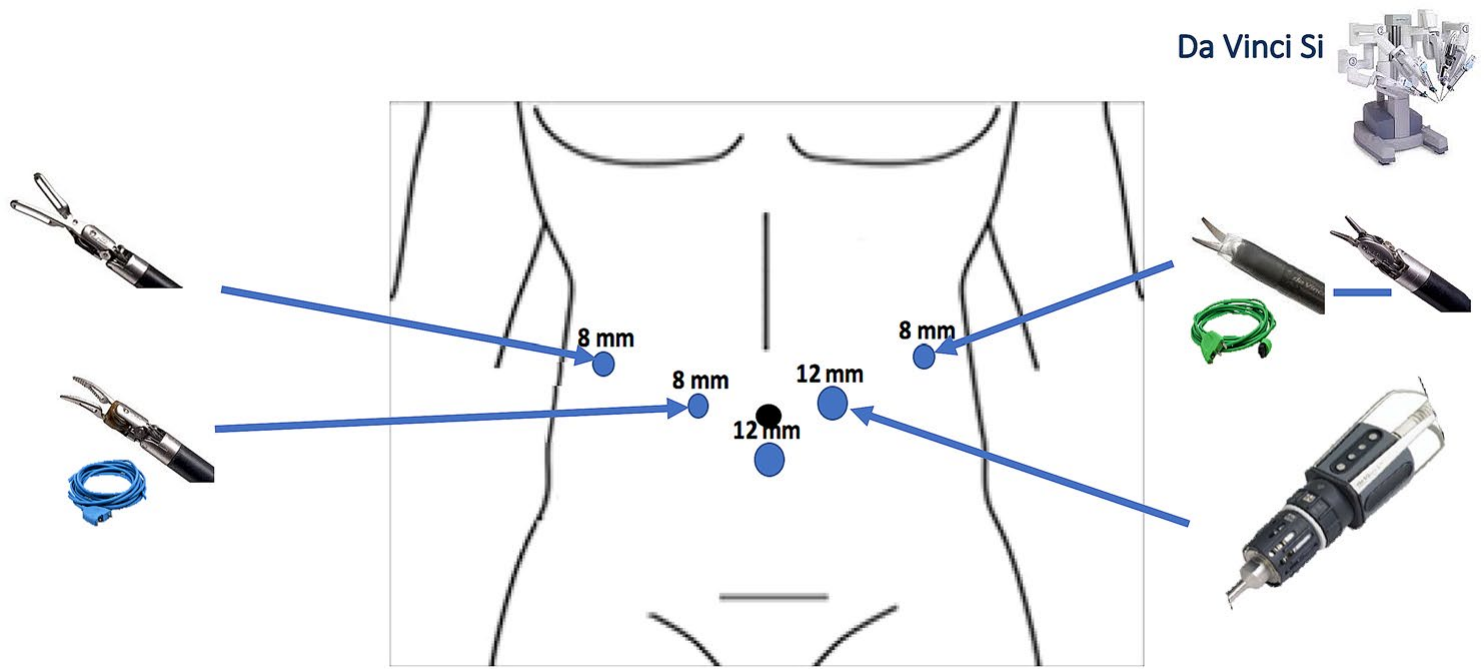
Trocars’ disposition and robotic instruments used for RDP with the da Vinci Si

**Fig. 2:**
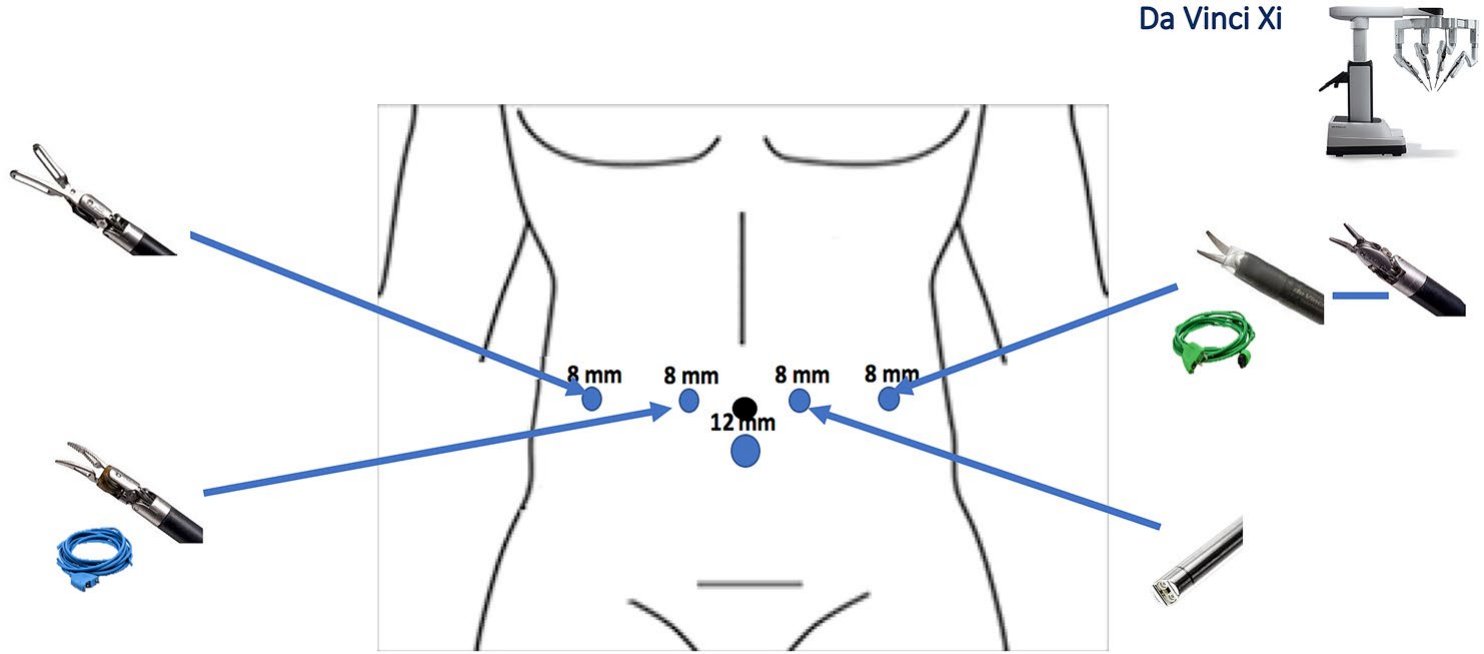
Trocars’ disposition and robotic instruments used for RDP with the da Vinci Xi

*Robotic DP* Patient position is similar to that of laparoscopic DP. For this approach, a five-port technique is adopted. The trocars disposition and the robot chart position change according to the type of robot used (Figs. 1 and 2). For dissection and retraction, monopolar scissor and Cadiere grasper are used, while the robotic energy devices employed are Gyrus PK SuperPulse Generator (Olympus, Center Valley, PA, USA) with the da Vinci Si or Maryland bipolar forceps (Intuitive Surgical, Sunnyvale, California, USA) during the last cases of the da Vinci Si or with the da Vinci Xi. The assistant’s trocar is then inserted and used for sutures insertion or suction. The surgical steps are similar to those of the laparoscopic approach. The pancreas is divided with robotic monopolar curved scissors and then the body of the pancreas is pulled up with the fourth arm to expose the posterior attachments of the organ. The special pulse-modulating robotic device (PKTM) or the Maryland bipolar forceps are used to seal all small tributary splenic vessels. The remnant pancreatic stump is predominantly oversewn with 4 or 5 interrupted sutures using robotic needle drivers with selective ligation of Wirsung duct. In case of thickness of the pancreas, the parenchyma is instead transected with a laparoscopic endostapler or with a robotic EndoWrist stapler for the da Vinci Xi. Once the gland is divided and fully freed from the attachments, the robot is undocked, and the specimen is placed in a plastic bag for a laparoscopic extraction through a suprapubic incision. Two drains are left near the pancreatic stump.

### Data collection

Preoperative data included age, gender, body mass index (BMI), American Society of Anesthesiology (ASA) score, preoperative diagnosis, intention to spleen preservation, and radiologic tumor size. Operative data included type of surgical procedure, operative time (OT), conversion rate, spleen preservation rate (calculated in those cases in which the spleen preservation was scheduled preoperatively on the basis of preoperative diagnosis), any additional organ resection (beyond splenectomy), pancreatic stump management (handsewn or stapler and number of charges used), and intraoperative complications. Postoperative data included LOS, both considering intensive care unit (ICU) and general ward, complications (according to the Clavien-Dindo Classification [20]), re-operation rate, and mortality. The surgical complications comprised surgical site infections, post-pancreatectomy hemorrhage [21], delayed gastric emptying [22], and post-operative pancreatic fistula (POPF) [23]. Pathological data included the final histology, tumor size, and the number of harvested lymph nodes.

### Costs analysis

Costs were valued using a micro-costing approach and expressed in euros and referred to 2020. Unit costs were collected from the accounting department of the hospital and divided in fixed costs and variable costs. In detail, fixed costs included the purchase and maintenance costs of the technology and the proportion of fixed costs attributable to a single intervention was estimated firstly deriving a cost per year on the basis of acquisition costs, the amortization period and annual maintenance costs, then dividing these costs for the overall number of interventions for which each technology was used. Overall variable costs (OVC) included items related to disposable instruments used within each intervention (consumable costs, CCs), operating room personnel (personnel costs, PCs), and length of stay, both ICU and general ward (hospital stay costs, HCs). For each intervention variable costs associated with the specific intervention were then estimated valuing resources used according to unit costs collected. Details about resources use and related costs are reported in Tables 1, 2, 3, 4 and 5.

**Table 1:** Details of fixed costs

	LDP	Si-RDP	Xi-RDP
Fixed costs attributable to the single intervention^*^ (€)	34.41	1583.75	1827.25

*LDP* laparoscopic distal pancreatectomy, *Si-RDP* robotic distal pancreatectomy with da Vinci Si, *Xi-RDP* robotic distal pancreatectomy with da Vinci Xi

*The costs are estimated dividing the acquisition costs for the amortization period, then considering annual maintenance costs and finally dividing annual costs by the overall number of interventions performed with the specific technology

**Table 2:** Personnel and hospital stay cost for MIDP

Personnel group	Unit cost (€/h)	Number of figures
Surgeon	62.69	2
Anesthetist	65.63	1
Anesthesiology technologist	27.96	1
Surgical nurse	30.50	1
Surgical technologist	31.34	1
Nurse assistant	21.36	1

	Unit cost (€/h)	
Hospital stay costs in a surgical ward	420.00	–
Hospital stay costs in an ICU	1150.00	–

*ICU* intensive care unit

**Table 3:** Consumable cost of LDP-group

	Unit costs (€)	Quantity	Overall CC (€)
5 mm laparoscopic trocar	36.60	1	36.60
12 mm laparoscopic trocar	48.00	1	48.00
Echelon 60	334.00	1	334.00
Echelon 45	298.00	1	298.00
Echelon 60 stapler charge	176.00	#	#
Echelon 45 stapler charge	190.00	#	#
Prolene suture	7.00	5	35.00
Verees needle	6.13	1	6.13
Ultracision	640.00	1	640.00

*CC* consumable cost

^#^On the basis of the number of laparoscopic charges used in each case

**Table 4:** Consumable cost of Si-RDP-group

	Unit costs (€)	Quantity	Overall CC (€)
Instrument arm drape	128.93	1	128.93
Camera arm drape	120.00	1	120.00
Camera head drape	117.02	1	117.02
Cadiere forceps	568.57	1	568.57
Maryland bipolar forceps	769.00	1	769.00
Monopolar curved scissors	909.73	1	909.73
Tip cover accessory	57.52	1	57.52
Cannula seal 8 mm	44.63	3	133.89
8 mm bladeless obturator	72.39	1	72.39
12 mm laparoscopic trocar	48.00	2	96.00
Robotic large needle driver	623.91	1*	623.91
Prolene suture	7.00	5	35.00
Laparoscopic stapler	219.00	1°	219.00
Laparoscopic stapler charge	189.00	#	#
Verees needle	6.13	1	6.13
Gelport	353.00	1^§^	353.00

*CC* consumable cost

*In case of handsewn closure of pancreatic stump

°In case of section of the pancreas with a stapler

^#^On the basis of the number of laparoscopic charges used in each case

^§^In case of conversion to Hand-Assisted Laparoscopic Surgery (HALS procedure)

**Table 5:** Consumable cost of Xi-RDP-group

	Unit costs (€)	Quantity	Overall CC (€)
Instrument arm drape	128.93	1	128.93
Column drape	61.53	1	61.53
Cadiere forceps	568.57	1	568.57
Maryland bipolar forceps	769.00	1	769.00
Monopolar curved scissors	909.73	1	909.73
Tip cover accessory	57.52	1	57.52
Cannula seal 5–8 mm	44.63	4	178.52
8 mm bladeless obturator	72.39	1	72.39
12 mm laparoscopic trocar	48.00	1	48.00
Robotic large needle driver	623.91	1*	623.91
Prolene suture	7.00	5	35.00
Laparoscopic stapler	219.00	1°	219.00
Laparoscopic stapler charge	189.00	#	189.00
Verees needle	6.13	1	6.13

*CC* consumable cost

*In case of handsewn closure of pancreatic stump

°In case of section of the pancreas with a stapler

^#^On the basis of the number of laparoscopic charges used in each case

Robotic staplers’ costs were excluded from the consumable cost analysis to avoid bias, as they were introduced only since 2016, and the choice to use them just depends on surgeon’s preference. In order to have three homogeneous groups, when the robotic staplers were used in the Xi-RDP-group, we considered the costs of the corresponding laparoscopic staplers and charges.

The study was approved by the Institutional review board.

### Statistical analysis

Categorical variables were depicted as number of cases and percentages, while continuous variables were expressed as mean ± (standard deviation) or median [25–75 percentile], depending on their distribution. Chi-square test and Fisher test were used to compare the distribution of categorical variables. For continuous variables, comparisons were made using independent *T*-test or Mann–Whitney test and multiple comparisons were performed by means of ANalysis Of Variance (ANOVA) or Kruskall-Wallis test. Post hoc comparisons were performed using Bonferroni or Mann–Whitney test with Bonferroni correction for p-value as appropriate.

Generalized linear models were used to evaluate the impact of the different surgical techniques on costs. In details, in addition to univariate analysis, multivariate models were developed including those variables whose *p* value < 0.10 at univariate analysis. In the multivariate analysis patients’ age was forced to enter while variables creating collinearity (on the basis of the variance inflation factor) were excluded. A *p*-value < 0.05 was considered statistically significant. All analyses were performed using Stata version 14.

## Results

Preoperative data are summarized in Table 6. No differences were reported in the demographical data. No statistically significant differences were reported in the preoperative diagnoses between the three groups, even if a higher percentage of cystic lesions was reported in the Si-RDP-group (62.9% in the Si-RDP vs 42.9% in the LDP-group and 34.3% in the Xi-RDP-group), while a higher percentage of pancreatic carcinoma was reported in the LDP-group and Xi-RDP-group (40% in the LDP-group and 37.1% in the Xi-RDP-group vs 17.1% in the Si-RDP-group).

**Table 6:** Preoperative data

	LPD-group	Si-RDP-group	Xi-RDP-group	*p* value
Age (years), mean ± SD	63.9 ± 16.9	60.4 ± 13.2	60.3 ± 14.5	0.527
Male:Female (%)	17:18 (48.6:51.4)	11:24 (31.4:68.6)	18:17 (51.4:48.6)	0.189
BMI (Kg/m^2^), mean ± SD	26.0 ± 5.5	26.2 ± 4.7	26.0 ± 4.4	0.988
ASA score, *n* (%)				0.927
ASA I	2 (5.7)	1 (2.9)	2 (5.7)	
ASA II	19 (54.3)	17 (48.6)	18 (51.45)	
ASA III	14 (40.0)	17 (48.6)	14 (40.0)	
ASA IV	0	0	1 (2.9)	
Preoperative diagnosis, *n* (%)				0.157
Pancreatic neoplasia	14 (40)	6 (17.1)	13 (37.1)	
Cystic lesion	15 (42.9)	22 (62.9)	12 (34.3)	
Neuroendocrine neoplasia	6 (17.1)	7 (20.0)	9 (25.7)	
Renal cell carcinoma pancreatic metastasis	0	0	1 (2.9)	
Dimension (cm), mean ± SD	4.0 ± 2.2	3.1 ± 2.0	3.5 ± 1.5	0.171

*BMI* body mass index, *ASA score* American society of anesthesiologists score

Perioperative data are summarized in Table 7. Although not statistically significant, the mean operative time was slightly lower in Xi-RDP-group (225 min) with respect to the Si-RDP-group (262 min), and to the LDP-group (247 min), (*p* = 0.155). Regarding the intraoperative variables, the conversion rate was significantly lower in the Si-RDP-group and Xi-RDP-group: 1/35 case (2.9%) and 0/35 cases (0%), respectively, versus 5/35 cases (14.3%) in the LDP-group (*p* = 0.045). The spleen preservation rate was higher in the RDP-groups, even if it did not reach a statistical significance (72.4% in the Si-RDP-group and 70% in the Xi-RDP-group vs 45% in the LDP-group, *p* = 0.171). In the RDP subgroups, the number of robotic instruments used was 4 in case of hand sewn closure of the pancreatic stump or 3 in case of the section of the pancreas with a stapler, with a mean number of robotic instruments used for each RDP of 3.3. No differences were reported in terms of overall post-operative complications rate between the three groups: 48.6% in the LDP-group, 57.1% in the Si-RDP-group and 48.6% in the Xi-RDP-group (*p* = 0.710). The incidence of complications with Clavien-Dindo ≥ III was similar between the three groups: 5.7% in the LDP-group, 2.9% in the Si-RDP-group and 8.6% in the Xi-RDP-group (*p* = 0.588). The length of hospital stay was nearly 1 day shorter in the RDP groups than the LDP group even if not statistically significant different: 11.1 ± 6.9 days in the LDP-group versus 9.5 ± 5.9 days in the Si-RDP-group and 9.3 ± 4.9 days in the Xi-RDP-group (*p* = 0.424). In the LDP-group, the length of hospital stay of converted cases was significantly longer than the cases treated with a minimally invasive approach: 15.6 ± 10.6 versus 9.8 ± 5.7 days, respectively (*p* = 0.039).

**Table 7:** Perioperative data

	LPD-group	Si-RDP-group	Xi-RDP-group	*p* value
Operative time (min), mean ± SD	247 ± 91	262 ± 87	225 ± 59	0.155
Type of operation, *n* (%)				0.027
Distal pancreatectomy	10 (28.6)	21 (60)	14 (40)	
Distal splenopancreatectomy	25 (71.4)	14 (40)	21 (60.0)	
Pancreas transection, *n* (%)				0.321
Hand-sewn	8 (25.3)	13 (38.2)	8 (24.2)	
Stapler	26 (76.5)	21 (61.8)	25 (75.8)	
Conversion, *n* (%)	5 (14.3)	1 (2.9)	0	0.045
Hand-assisted laparoscopic surgery	1 (2.9)	1 (2.9)	0	
Open surgery	4 (11.4)	0	0	
Spleen preservation rate, *n* (%)	9/20 (45%)	21/29 (72.4%)	14/20 (70%)	0.171
Overall complications, *n* (%)	17 (48.6)	20 (57.1)	17 (48.6)	0.710
POPF, *n* (%)	14 (40.0)	12 (34.5)	11 (31.4)	0.747
BL	11 (31.4)	10 (28.6)	5 (14.3)	
Grade B	3 (8.6)	2 (5.7)	6 (17.1)	
Grade C	0	0	0	
Abdominal collection, *n* (%)	3 (8.6%)	2 (5.7%)	4 (11.4%)	0.694
Clavien–Dindo score ≥ III, *n* (%)	2 (5.7)	1 (2.9)	3 (8.6)	0.588
Reoperation, *n* (%)	0 (0%)	0 (0%)	0 (0%)	1
Length of hospital stay, mean ± SD (days)	11.1 ± 6.9	9.5 ± 5.9	9.3 ± 4.9	0.424
ICU recovery, *n* (%)	5 (14.3)	2 (5.7)	3 (8.6)	0.461
In hospital mortality, *n* (%)	0 (0%)	1 (2.9%)	0 (0%)	1

*POPF* post-operative pancreatic fistula, *BL* biochemical leak, *ICU* intensive care unit

Analyzing the pathological data, no differences were detected between the pathological diagnoses (Table 8). A significant difference was reported in the mean number of harvested lymph nodes, with a lower number reported in the LDP-group: 10.6 ± 8.1 vs 14.2 ± 13.7 in the Si-RDP-group and 23.2 ± 16.7 in the Xi-RDP-group (*p* = 0.001).

**Table 8:** Pathological data

	LPD-group	Si-RDP-group	Xi-RDP-group	*p* value
Pathological diagnosis, *n* (%)				0.065
PDAC	8 (22.9)	2 (5.7)	9 (25.7)	
Adenocarcinoma	0	3 (8.6)	1 (2.9)	
IPMN	2 (5.7)	6 (17.1)	2 (5.7)	
MCN	4 (11.4)	6 (17.1)	1 (2.9)	
SCN	8 (22.9)	4 (11.4)	3 (8.6)	
NET	9 (25.7)	10 (28.6)	9 (25.7)	
SPT	1 (2.9)	0	4 (11.4)	
RCC metastasis	0	0	1 (2.9)	
Other	3 (8.6)	4 (11.4)	5 (14.3)	
Tumor size (mm), mean ± SD	39.3 ± 23.8	28.4 ± 19.6	33.1 ± 12.4	0.074
Harvest lymph nodes, mean ± SD	10.6 ± 8.1	14.2 ± 13.7	23.2 ± 16.7	0.001

*PDAC* pancreatic ductal adenocarcinoma, *IPMN* intraductal papillary mucinous neoplasm, *MCN* mucinous cystic neoplasm, *SCN* serous cystic neoplasm, *NET* neuroendocrine tumor, *SPT* solid pseudopapillary tumor, *RCC* renal cell carcinoma

The results of the costs’ analysis are summarized in Table 9. No differences were reported about the personnel costs and the hospital stay costs between the three groups (*p*-value = 0.132 and *p* = 0.069, respectively). The comparison of the consumable costs showed significant higher costs of the Si-RDP-group and the Xi-RDP-group respect to the LPD-group (median values being €3434 and €3409 versus €1505, respectively, *p* < 0.05). Overall variable costs and overall costs including fixed costs of the Si-RDP-group and the Xi-RDP-group resulted significantly higher respect to the LPD group: median values being €7856 and €7981 versus €6968, and €9440 and €9809 versus €7002, respectively (*p* < 0.05). However, at multivariate analysis, adjusting for age, ASA risk score and spleen preservation, overall variable costs were no more significantly different between the LPD-group and the Xi-RPD-group (*p* = 0.099) (Tables 10 and 11). Moreover, when distinguishing for the intention to spleen preservation on the basis of the preoperative diagnosis, the multivariate analysis revealed that the overall variable costs were no longer significantly different between the LPD-group versus the Si-RPD-group and the Xi-RPD-group in the subgroup of patients in which the spleen preservation was indicated (*p* = 0.115 and *p* = 0.261, respectively) (Table 11).

**Table 9:** Costs’ analysis

	LPD-group	Si-RDP-group	Xi-RDP-group	*p* value	*p* value
Personnel’s costs (€), median [Q1–Q3]	1143 [957–1385]	1234 [1108–1612]	1183 [907–1360]	0.132	
Hospital stay costs (€), median [Q1–Q3]	4200 [2940–5460]	2940 [2520–4200]	3360 [2520–4620]	0.069	
Consumables costs (€), median [Q1–Q3]	1505 [1151–1772]	3434 [3061–3646]	3409 [3201–3579]	< 0.001	< 0.05 LDP-group vs Si-RDP-group < 0.05 LDP-group vs Xi-RDP-group
Overall variable costs (€), median [Q1–Q3]	6968 [5961–8392]	7,856 [7117–9754]	7981 [7095–9040]	0.016	< 0.05 LDP-group vs Si-RDP-group < 0.05 LDP-group vs Xi-RDP-group
Overall costs (€), median [Q1–Q3]	7002 [5996–8426]	9440 [8700–11338]	9809 [8922–10867]	< 0.001	< 0.05 LDP-group vs Si-RDP-group < 0.05 LDP-group vs Xi-RDP-group

**Table 10:** Univariate analysis

	Coef (Std.Err)	*p* value
Overall variable costs		
Age	0.003 (0.002)	0.116
Male gender	0.01 (0.06)	0.855
Body mass index	0.01 (0.01)	0.107
ASA risk score	0.16 (0.05)	0.002
Benign tumor	− 0.04 (0.07)	0.543
Spleen preservation	− 0.15 (0.06)	0.012
Conversion	− 0.07 (0.13)	0.628
Overall costs		
Age	0.002 (0.002)	0.264
Male gender	0.002 (0.06)	0.97
Body mass index	0.01 (0.01)	0.135
ASA risk score	0.15 (0.05)	0.003
Benign tumor	− 0.05 (0.06)	0.422
Spleen preservation	− 0.10 (0.06)	0.086
Conversion	− 0.16 (0.13)	0.205

**Table 11:** Multivariate analysis

	Coef (Std.Err)	*p* value
Overall variable costs		
LDP-group vs Si-RDP-group	0.18 (0.07)	0.016
LDP-group vs Xi-RDP-group	0.11 (0.07)	0.099
Overall costs		
LDP-group vs Si-RDP-group	0.33 (0.06)	< 0.001
LDP-group vs Xi-RDP-group	0.31 (0.06)	< 0.001

	No intention spleen preservation		Intention spleen preservation	
	Coef (Std.Err)	*p* value	Coef (Std.Err)	*p* value
Overall variable costs				
LDP-group vs Si-RDP-group	0.25 (0.13)	0.058	0.14 (0.09)	0.115
LDP-group vs Xi-RDP-group	0.20 (0.09)	0.037	0.11 (0.10)	0.261
Overall costs				
LDP-group vs Si-RDP-group	0.40 (0.12)	0.001	0.30 (0.08)	< 0.001
LDP-group vs Xi-RDP-group	0.39 (0.08)	< 0.001	0.30 (0.09)	0.001

## Discussion

The economic impact of the robotic approach on pancreatic surgery, and particularly on distal pancreatectomy, is still under debate, with controversial published studies [3, 9–14]. Indeed, although the majority of authors reported higher costs associated to RAS with respect to DML [11, 24], some evidences underlining the limitations and biases of the majority of the cost-analysis published so far, have opened new perspectives on this specific issue [6, 15].

Therefore, as from a clinical point of view RDP seems to be associated with some positive impact on the outcomes with respect to the LDP, it is still a matter of debate whether the use of the da Vinci is sustainable, and whether the advantages of the da Vinci Surgical System can justify its use for distal pancreatectomy.

For these reasons, to evaluate the costs-effectiveness of RAS and DML in this setting, different factors to be evaluated comprise both those strictly related to the instrumentation and its use and those referring to the clinical outcomes. Thus, to perform our structured cost-analysis, we divided the costs in fixed and variable costs, including in the first the purchase and maintenance of the technology, and in the latter the disposable instruments (consumable costs), the length of stay and the post-operative course (hospital stay costs), and the operating room personnel costs (personnel costs) which are associated to the OT.

Interestingly, despite our costs’ analysis confirmed that the overall cost including fixed costs and overall variable costs of RDP resulted higher compared with that of the LDP, at univariate and multivariate analysis, the overall variable costs were no longer significantly different between the LPD-group and the Xi-RPD-group. In our opinion, this data can be explained by the standardization of the technique, the perioperative outcomes, and by a discount of robotic instruments’ purchase costs coming from the multidisciplinary high-volume center setting as well.

In fact, the standardization of the technique with the improvement in robotic expertise, allows to optimize the use of instruments, by performing all phases of the surgical operation with an average of only 3.3 robotic instruments, therefore contributing to minimize the CCs [13]. Indeed, in our series we used a basic set for each surgical approach including the essential instruments to perform the operation, and all other instruments were used on request, only if strictly necessary. In detail, in the robotic approach, we standardized the use of the monopolar scissors on the right hand and of the Maryland bipolar forceps in the left hand for dissection, and of the Cadiere grasper for retraction in the fourth arm, while a fourth instrument was used only in selected cases when suturing was needed. Instead, by laparoscopy we always used an energy device for dissection.

Among the perioperative outcomes, OT, conversion rate, and LOS are the main key factors that might have had a major impact on costs. Indeed, in our series, the reported shorter mean operative time of the Xi-RDP-group with respect to Si-RDP-group and LDP-group, even if not statistically significant, has played a role in reducing the PCs, in line with similar works already reported also for other indications [15]. In fact, the reduced docking time of the da Vinci Xi with respect to the da Vinci Si, as well as the improvement of the surgical workflow, represent two well consolidated advantages of the latest version of the da Vinci System, that can independently influence the OT and therefore, indirectly, also the costs. In our series, several factors might contribute to explain the reduction of operative time and PCs of Xi-RDP also in comparison with LDP, firstly the higher experience with RAS [15], and secondly the advantages of RAS over DML that simplify the execution of all the difficult tasks of LDP, such as dissection of the pancreatic body and tail from splenic vessels, suturing, control of bleeding, completion of the retropancreatic tunnel, and suturing the pancreatic stump with the closure of Wirsung duct [25].

Other important perioperative factors influencing costs in our series, as well as in previous papers [6, 24, 26–30], are the conversion rate and the LOS. In particular, the significantly lower conversion rate of RDP that we reported, index assessing the ability to deal with the complexity of the procedures and complete them with a minimally invasive approach thanks to the greater dexterity and precision of robotic manipulation in vessel dissection and bleeding control [29], could partially justify also the shorter LOS in the RDP groups, that resulted nearly 1 day shorter than the LDP group. In fact, the LOS of patients that had undergone LDP completed with the laparoscopic approach was significantly lower than the LOS of converted cases of LDP-groups, while it was similar to the LOS of RDP groups.

In this respect, it has also to be noticed that, despite the minimally invasive approach and the relatively low incidence of complications, we reported a mean LOS longer as compared with other robotic and laparoscopic case series of international literature. However, our data are comparable with the analysis conducted in an Italian multicenter cohort of 236 patients that had undergone RDP [31], and this could probably be due to cultural factors with a more cautious policy used for discharging patients from hospital, as in Italy patients expect to leave hospital only when fully recovered and therefore needing little outpatient [32]. As a result, these data suggest that the possible optimization of costs could theoretically be even greater.

Another aspect which deserves to be highlighted among the positive impact of the RAS versus the DML on the outcomes of the distal pancreatectomy, although difficult to quantify within a cost analysis, is the higher spleen preservation rate. In this regard, our data are in line with literature [2, 7, 17, 33], as the greater effectiveness of robotic system in controlling splenic vessels bleeding resulted in a higher spleen preservation rate associated with RDP among patients scheduled for spleen preservation with respect to LDP. This outcome is important not only for the biological benefit of spleen preservation, but also as the reduced use of stapler and stapler charges, necessary for the section of the splenic vessel in case of splenectomy, could contribute to reduce the CCs of RDP.

Interestingly, in this regard, the multivariate analysis of our costs’ analysis revealed that the overall variable costs were no longer significantly different between LPD and RPD, both for the da Vinci Si and the da Vinci Xi, in the subgroup of patients in which the spleen preservation was indicated on the basis of the preoperative diagnosis. These data suggest that the use of the da Vinci Surgical System, both Si and Xi, could be particularly indicated in those cases in which the spleen preservation is scheduled.

From a histological standpoint, we reported a higher percentage of cystic lesions in the Si-RDP-group, while on the contrary a higher percentage of pancreatic carcinoma was reported in the Xi-RDP-group, explaining also the higher number of lymph nodes harvest in the Xi-RDP-group. In fact, while in the initial experience with RAS we preferred to treat benign lesions, with the overcoming of the learning phase with the robotic technology, more and more complex cases were treated with RDP, particularly since the introduction of the da Vinci Xi. We acknowledge that this aspect could introduce a bias, but it is interesting to note that although the complexity of cases has increased over time within the RDP whole group, the outcomes of patients belonging to the Xi-RDP-group were not worse respect to those of the Si-RDP-group and, in some cases, resulted even better. Furthermore, although the number of pancreatic malignant tumors was similar between LDP-group and Xi-RDP-group, the number of harvested lymph nodes was significantly higher in the latter, probably as a consequence that, because of increasing confidence with the robotic system acquired over time, as well as the improvement offered by the Xi version, more complex cases of malignant tumors located in the isthmus or central part of the body of the pancreas needing a more extended lymphadenectomy, were treated with a minimally invasive technique. This is a crucial aspect, to encourage the use of robot, particularly if included in a setting of possible optimization of costs. Indeed, even though the use of robotic technology to perform a robotic DP was first reported by Melvin in 2003 [34], so far the use of minimally invasive technique is limited to highly specialized centers, while the open approach is still chosen in many surgical center. Actually, the technical advantages of robotic systems could allow the expansion of the adoption of minimally invasive surgery in the field of pancreatic surgery [35], without impairing the clinical outcomes, and, therefore, on the cost-effective management of patients that require a DP, the higher possibility to offer a minimally invasive approach also for malignant and more complex tumors might be increasingly taken in consideration. This could be particularly important also for the LOS reduction and the faster return to normal daily activities, making more likely a shorter time between surgery and adjuvant chemotherapy compared to an open DP [36]. Although this data can be difficult to be evaluated in economic terms, we think that it should be taken into consideration when analyzing the right balance between these advantages and the higher costs of RDP.

Our study has some limitations. First of all, its retrospective nature, the heterogeneity of the sample and the not standardized choice of the laparoscopic versus robotic approach. However, using a case–control matching analysis we tried to mitigate these issues by obtaining three comparable groups. Another criticism might be the exclusion of robotic staplers’ costs to avoid bias in the analysis of consumable costs. Indeed, as previously declared, at the time our Si interventions were performed, they were not available for that robotic platform. Furthermore, so far there is no evidence in literature of the superiority of the endo-wristed staplers respect to the laparoscopic ones, and the use of anyone of them during an RDP is merely a surgeon’s choice. Therefore, considering the costs of endo-wristed staplers during the analysis would have introduced a bias. For this reason, in order to have three homogeneous groups, when the robotic staplers were used in the Xi-RDP-group we considered the costs of the corresponding laparoscopic staplers and charges, as they were always usable in all cases. Moreover, since this study comes from a high-volume center for pancreatic surgery and also for RAS, the results of the analysis may not be applicable to all centers, also because the cost-analysis was performed using specific resources use and economic data of the accounting department of our hospital and it could be difficult to generalize these data to other parts of the world. In this regard, although currently difficult to define a threshold, the use of the da Vinci Surgical System in a high-volume RAS center, can contribute itself to the reduction of the fixed costs, by reducing the depreciation charge, and of each operation OVCs of RDP thanks to reduction of the CCs. Indeed, in our experience, thanks to the high number of robotic operations performed each year at the Multidisciplinary Center of Robotic Surgery at Cisanello Hospital in Pisa, we obtained a 2% discount of robotic instruments’ purchase costs, partially contributing to reduce the gap of CCs between the LDP and RDP. Moreover, performing even more than a thousand robotic interventions per year, as we have experienced at our Centre, thanks to the purchase of a large number of robotic instruments it is possible to obtain a further bulk discount, thus reducing the average cost of robotic instruments for each robotic intervention, with the possibility to obtain a further reduction of CCs of RDP. This represents another reason, in addition to those mentioned above, for further encourage the wide spreading of robotic technologies in the field of pancreatic surgery. Therefore, it may well be crucial to encourage the spread of multidisciplinary high-volume centers, in which surgeons coming from different hospitals can have access with the opportunity to operate on their own patients, benefiting from the most advanced technologies and also reducing costs at the same time. This could be one of the key points to make sustainable the use of robots for surgical operation, particularly for more complex cases, such as those involving pancreatic surgery. Furthermore, of note that according to accounting standards fixed costs attributable to the single intervention were estimated considering the 5-year amortization period of the instruments. This period is obviously limited when compared to the actual period of use of the technologies, thus spreading costs over the entire product life cycle can give a more realistic picture of the economic impact and in this case the gap in fixed costs between LDP and RDP would be reduced. Finally, this study is a cost analysis and further studies properly evaluating the cost-effectiveness of RDP, also including an assessment of the quality of life and the impact of the procedure on long-term outcomes, would shed light on the cost-effectiveness of RDP vs LDP.

In conclusion, RAS is more expensive than DML for DP because of higher acquisition and maintenance costs. The flattening of these differences considering only the variable costs suggests a possible optimization of the cost-effectives of RAS, particularly in high-volume centers. As a result, the advantages of robotic technology could be encouraged in this setting, as it can favor the use of minimally invasive approach for DP by a larger cohort of pancreatic surgeons and expand the field of this approach to more complex pancreatic lesions, so far treated by open surgery. Moreover, RAS might be particularly indicated for MIDP when the spleen preservation is scheduled.
